# Sexual dimorphism and skull size and shape in the highly specialized snake species, *Aipysurus eydouxii* (Elapidae: Hydrophiinae)

**DOI:** 10.7717/peerj.11311

**Published:** 2021-04-20

**Authors:** Bartosz Borczyk, Łukasz Paśko, Jan Kusznierz, Stanisław Bury

**Affiliations:** 1 Department of Evolutionary Biology and Conservation of Vertebrates, University of Wroclaw, Wrocław, Poland; 2 Department of Comparative Anatomy, Institute of Zoology and Biomedical Research, Jagiellonian University, Kraków, Poland

**Keywords:** Sexual dimorphism, Allometry, Skull, Specialization, Feeding niche, Foraging

## Abstract

**Background:**

Snakes exhibit sexual dimorphism in both head size and shape. Such differences are often attributed to different reproductive roles and feeding habits. We aim to investigate how sexual dimorphism is displayed in the highly specialised fish-egg-eating snake, *Aipysurus eydouxii*, by analysing two complementary features: body size and skull morphology.

**Methods:**

We used data on body length, weight, and skull shape from 27 measurements of 116 males and females of *A. eydouxii*. We investigated both sexual dimorphism and allometric (multivariate and bi-variate) properties of skull growth in the analysed data set.

**Results:**

We found that although there was female-biased sexual size dimorphism in body length, females were not heavier than males, contrary to what is commonly observed pattern among snakes. Moreover, females tend to possess relatively smaller heads than males. However, we only found very subtle differences in skull shape reflected in nasal width, mandibular fossa, quadrate crest and quadrate length.

**Discussion:**

We suggest that the feeding specialisation in *A. eydouxii* does not allow for an increase in body thickness and the size of the head above a certain threshold. Our results may be interpreted as support for prey-size divergence as a factor driving skull dimorphism since such species in which the sexes do not differ in prey size also shows very subtle or no differences in skull morphology.

## Introduction

The differences between sexes have attracted the attention of biologists for decades; not only do they refer to reproductive investments or sex-related traits, but consequences of differential reproductive output can be observable among many other features (Darwin, 1871). One of the most visible manifestations of sex-biased differentiation concerns body size, either on the scale of the entire organism or its parts (e.g. Berns, 2013). Sexual size dimorphism has been widely studied in different taxa (e.g. Fairbairn, 1997; Berns, 2013). One group that has been extensively studied is snakes due to distinct differences between the sexes that are visible in numerous species (Shine, 1993). For example, Arafura File Snakes (*Acrochordus arafurae*) reaching extremes with females being up to 10 times heavier than males (Shine, 1991). Sexual size dimorphism is followed by differentiated energy requirements that can manifest as food intake and type or size of prey (Elgee & Blouin-Demers, 2011; Borczyk, 2015). The latter aspect is of specific interest since, in many instances, it requires adjustment of not only ecological properties (like habitats, preferences towards appropriate food resources, or feeding niche divergence) but also of morphology (Camilleri & Shine, 1990). Since snakes are legless and cannot divide their prey into pieces (except certain species like *Fordonia leucobalia* and *Gerarda prevostiana*; see Jane, Voris & Ng, 2002), most morphological adaptations related to feeding (from capturing prey to swallowing it) concerns the head and skull (e.g. Cundall & Greene, 2000). The snake skull has been the subject of numerous studies, but most researchers focused on overall morphology and structure (reviewed by Cundall & Greene, 2000; Cundall, Irisch & Morphology, 2008). Only a few studies focused on allometric changes or sexual dimorphism in the snake skull form (Cundall & Greene, 2000; Camilleri & Shine, 1990; Murta-Fonesca & Fernandes, 2016; Andjelković, Tomović & Ivanović, 2016). Thus, the existence, degree, and origin (e.g., is dimorphism a result of an allometric growth pattern or is its degree constant during ontogeny?) of sexual dimorphism in snake skull size and shape remain largely unknown.

So far, most studies on snake skull allometry and sexual dimorphism have focused on species that feed on a relatively large spectrum of prey; moreover, they have been restricted to a few snake lineages, such as Natricinae, Xenodontinae, or Crotalinae (e.g. Rossman, 1980; Young, 1989; Hampton & Kalmus, 2014; Andjelković, Tomović & Ivanović, 2016; Hampton & Moon, 2013). Under such circumstances, intraspecific food-niche partitioning followed by morphological adjustments are easily predictable, namely the larger sex, in response to elevated food requirements, increases the size of its skull to ingest larger prey (Elgee & Blouin-Demers, 2011). It is more difficult to explain a scenario where strong feeding specialisation is equalised between males and females and the size of ingested food particles does not change. *Aipysurus eydouxii* is such an example, as it is one of the few snake species that feed only on fish eggs (Voris & Voris, 1983). A diet composed of numerous, but small items, do not impose problems with swallowing because the prey items are much smaller than the maximum gape size of even a small snake.

Here we aim to investigate how sexual dimorphism is displayed in a highly specialised snake species, *A. eydouxii*, by analysing two complementary features: body size and skull morphology. We predict female-biased sexual size dimorphism, which is the most common pattern among viviparous species. Moreover, because there are no prey size differences between the sexes, and the mating behaviour of sea snakes does not involve biting or other interactions in which head size would be important, we assume that there should be no dimorphism in skull size and shape.

The Marbled Sea Snake (*Aipysurus eydouxii*) is member of Hydrophiinae and an elapid radiation of both terrestrial and fully marine snakes. It can grow up to 100 cm in total length. It is a viviparous snake living at depths of up to 30–50 m in turbid waters (Heatwole, 1999). It feeds almost exclusively on fish eggs (Voris & Voris, 1983). Moreover, an evolutionary shift from hunting fishes to feeding on their eggs is reflected in a 50- to 100-fold decrease in the venom toxicity compared to its closest relatives (there is no need to immobilise struggling prey), atrophied venom glands, and loss of effective fangs (Tu, 1974; Gopalakrishnakone & Kochva, 1990; Li, Fry & Kini, 2005). Adaptation to fish-egg feeding also resulted in a decrease in body size (compared to its relatives), reduction and loss of teeth, strong throat musculature (suction), and fusion of lip scales (McCarthy, 1987).

## Materials and Methods

**Figure 1:**
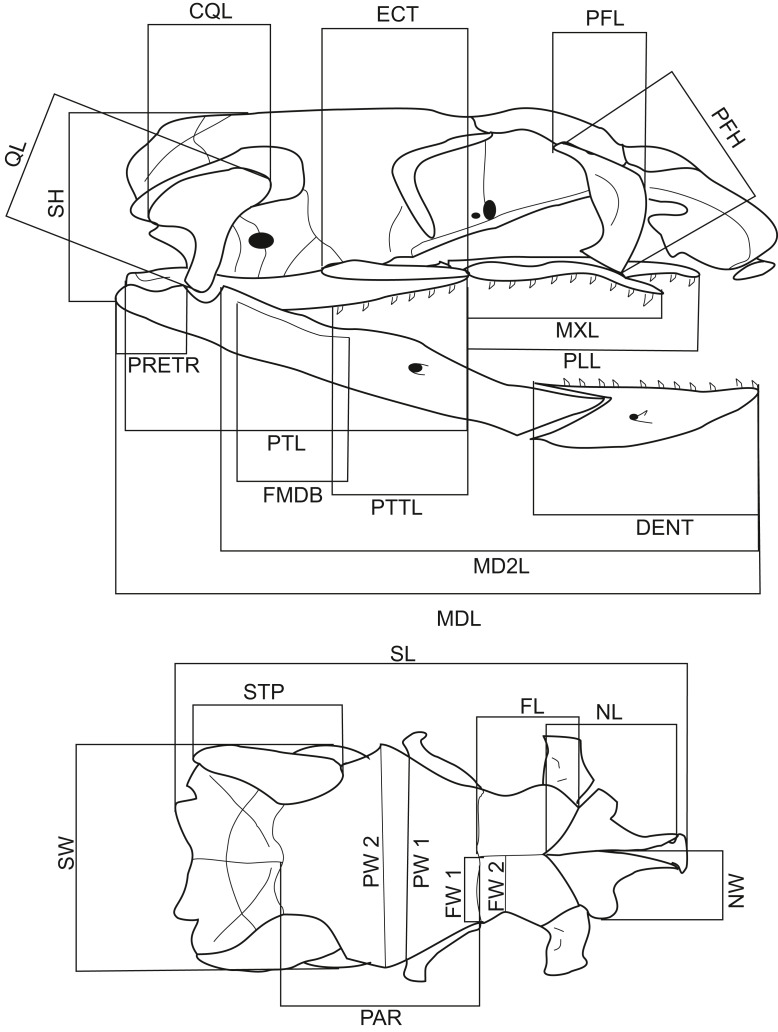
Lateral and dorsal views of a *Aipysurus eydouxii* skull. Diagrams of lateral and dorsal views of a *Aipysurus eydouxii* skull to show osteological measurements. Abbreviations: CQL, quadrate crist length; DENT, length of the dental bone; ECT, length of the ectopterygoid bone; FL, frontal length; FMDB, mandibular fossa length; FW1, frontal width at the fronto-parietal suture; FW2, frontal width at the narrowest point; MDL, length of the mandibula; MD2L, length of the in-levar of the mandibula; MXL, length of the maxilla; NCL, nasal component length; NL, nasal length; NW, nasal width; PAR, parietal length; PFH, prefrontal bone height; PLL, palatine length; PMW, width of the premaxillary bone; PRETR, length of the retroarticular proces; PTL, pterygoid length; PTTL, pterygoid tooth row length; PW1, parietal width at the postorbital articulation; PW2, parietal width at the widest point of the parietal bone; QL, quadrate bone length; SH, skull height, STP, length of the supratemporal bone; SW, skull width.

A total of 116 dry snake skulls of *A. eydouxii* (46 males and 70 females) from the collection of the Field Museum (Chicago, IL, USA) were examined. Of these 116 specimens, the snout-to-vent length (SVL) was recorded in 48 (26 males, 22 females) and the body weight, (BW) in 43 (25 males, 18 females). The body weight was taken prior to fixation. For each skull, 26 measurements were taken (Table 1, Fig. 1). These distances were chosen as representative of the overall skull shape and the proportions of skull elements involved in feeding. All measurements were taken with digital calliper directly from the skull.

**Table 1:** List of skull measurements of *Aipysurus eydouxii*.

Abbreviation	Measurement
CQL	Quadrate crest length
DENT	Dentary length
ECT	Ectopterygoid length
FL	Frontal length
FMDB	Mandibular fossa length
FW1	Frontal width taken at fronto-parietal contact
FW2	Frontal width taken at its narrowest point
MD2L	Mandible length taken from the rostral tip of the mandible to the mandible joint
MDL	Mandible length taken from it rostral tip to the caudal tip
MXL	Maxilla length
NCL	Nasal component length taken at naso-frontal articulation to the most rostral tip of premaxilla
NL	Nasal length
NW	Nasal width
PAR	Parietal length
PFH	Prefrontal height
PFL	Prefrontal length
PLL	Palatine length
PRETR	Retroarticular proces length
PTL	Pterygoid length
PTTL	Length of tooth row on pterygoid
PW1	Parietal width at postorbital process
PW2	Parietal width
QL	Quadrate length
SH	Skull height
SL	Skull length
STP	Supratemporal length
SW	Skull width

### Statistical analysis

In order to find size-free data patterns of inter-sex variation, we conducted a MANOVA analysis on size free data. According to Allometric Burnaby’s Method (Bookstein, 1991; Reyment, 1991; Rohlf, 2010), the effect of body size and ontogenetic allometry of the specimens was removed from the data matrix by the projection of the character set on the plane orthogonal to the size vector (the first eigenvector (PC I) from the variance-covariance matrix of log_10_-transformed data). A Tukey’s HSD post-hoc test was used to test for unequal N. To estimate the size effect between group means for statistically significant variables, the Cohen’s d Coefficient was calculated (Cohen, 1988; Szymczak, 2015). The Cohen’s d coefficient values beetwen d = 0.2 and d = 0.49 is considered a weak effect, d = 0.5 to d = 0.79 is an average effect, and d > 0.8 denotes a strong effect, however it should be interpreted with caution.

The number of variables was also reduced using multidimensional methods: Canonical Variate Analysis (CVA) and Principal Component Analysis (PCA). Both analyses were based on the variance-covariance matrix. Canonical Variate Analysis is a more appropriate method due to the minimalisation of the ratio of intra-group to inter-group variance. However, it requires assumptions of normal distributions of characteristics within groups and homogeneity of their variances. Principal Component Analysis does not consider intra-group variance; it only maximises the individual variance and does not require additional assumptions. Normality of the distribution of characters was tested with the Shapiro-Wilk’s test and the homogeneity of variance with Levene’s test.

### Multivariate and bivariate allometry

The multivariate allometric coefficient represents the growth pattern of a trait in respect to the overall size and differs from simple bivariate allometry coefficient, which focuses on two-traits relationship, with usually representing a size measure (i.e., length, weight, surface).

The loadings on the first eigenvectors (PC I) from the variance-covariance matrices of the log_10_-transformed data for each sex can be interpreted as multivariate allometric coefficients (Strauss, 1987; Rohlf, 1998; Reyment, 1991; Bookstein, 1991). PC I is a descriptor of variability which stems from the biggest source of variation in the group (Rohlf, 1998). This is most frequently the variability of body size. The loadings were rescaled (average loading = 1, full isometry) according to Strauss & Bookstein (1982).

The characters that were statistically different between the sexes were subjected to further analysis to determine if the divergence between the sexes was a function of allometric growth or had a different origin. The analysis of allometry was performed on log-transformed measurements. The slopes for skull size (skull length SL, skull height SH, skull width SW) were calculated with snout to vent length (SVL) and body weight (BW) as the baselines (the “snake size” variables), and other sexually dimorphic skull measurements were scaled against SL. Because both dependent and independent variables are biased by measurement error, a Reduced Major Axis Regression (RMA) was performed (Sokal & Rohlf, 1995). The slopes were tested for divergence from 1 in the case of linear measurements and 3 when BW was scaled against SVL, which would indicate negative or positive allometry, and the slopes were compared between the sexes.

All calculations were conducted using Microsoft Excel, NTSYS 1.8 (Rohlf, 1996), NTSYS 2.21 (Rohlf, 2010), R 3.6.1 (The R Foundation for Statistical Computing, 2019) and STATISTICA 13 (TIBCO Software Inc, 2017) and RMA: Software for Reduced Major Axis Regression (Bohonak & Van der Linde, 2004).

## Results

### Assumptions

The Levene’s Test indicates that the hypothesis of homogeneity of variance was only violated for three variables (6, 11, 25). The Shapiro–Wilk’s test showed that for most empirical distributions, the hypothesis of a normal distribution could not be rejected (52 empirical distributions, of which 22 were not normal). Since the distributions of characters and homogeneity of variance were compatible, in most cases, with the assumptions, MANOVA and canonical variates analyses were performed.

### Sexual size dimorphism

**Figure 2:**
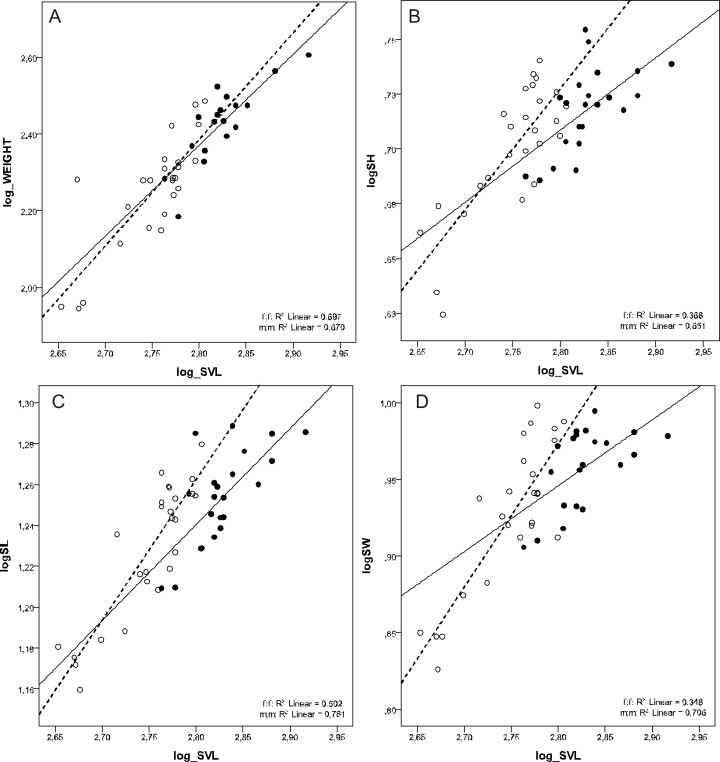
Scatterplots of log-transformed BW, SH, SL and SW against the log-transformed SVL of males (open circles) and females (filled circles) of *Aipysurus eydouxii*. Scatterplots of (A) log-transformed body weight (log_WEIGHT) against the log-transformed snout to vent length (log_SVL), (B) log-transformed skull height (log_SH), (C) log-transformed skull length (log_SL) the log-transformed snout to vent length (log_SVL) and (D) log-transformed skull width (log_SW) against the log-transformed snout to vent length (SVL) of males (open circles) and females (filled circles) of *Aipysurus eydouxii*.

There was a female-biased sexual dimorphism in SVL (F_1, 46_ = 49.122, *p* < 0.001) and BW (F_1, 41_ = 22.726, *p* < 0.001). However, there was no sexual dimorphism in BW when corrected for SVL (ANCOVA test F_1, 41_ = 0.244, *p* = 0.624). MANCOVA also showed that males had relatively longer, higher, and wider skulls than females at the same SVL (Wilks’ ƛ = 0.772, F_3,42_ = 4,127, *p* = 0.012) (Tables 2 and 3, Fig. 2).

**Table 2:** Descriptive statistics for *A. eydouxii*.

Character	Males	Females
SVL	564.5 ± 53.941	675.9 ± 55.914
	450–640	580–825
	*N* = 26	*N* = 22
BW	181.88 ± 63.487	274.2 ± 60.377
	40–306	153–404
	*N* = 26	*N* = 18
SL	17.03 ± 1.172	17.62 ± 0.951
	14.44–19.04	15.18–19.44
	*N* = 46	*N* = 69
SH	5.03 ± 0.3	5.15 ± 0.25
	4.21–5.55	4.51–5.68
	*N* = 46	*N* = 69
SW	8.51 ± 0.794	8.93 ± 0.621
	6.7–9.96	7.31–10.19
	*N* = 44	*N* = 68

**Note:**

Descriptive statistics (mean±standard deviation, min-max and sample size) for snout-to-vent lenght (SVL), body weight (BW), skull lenght (SL), skull height (SH) and skull width (SW) of male and female *Aipysurus eydouxii*. All measurements are in milimiters except of body weight, which is given in gramms.

**Table 3:** Results of MANCOVA.

Variable	SS	df	MS	F	*p*
logSL	0.003	1	0.003	90.783	0.003
logSH	0.002	1	0.002	70.200	0.01
logSW	0.004	1	0.004	60.381	0.015

**Note:**

Results of MANCOVA test for the differences in log-transformed skull length (SL), width (SW) and height (SH) with the snout-vent length (SVL) as a covariate between males (*N* = 26) and females (*N* = 21) of *Aipysurus eydouxii*.

### MANOVA

The differences between males and females were statistically significant (Wilks Multivariate Test of Significance, *p* = 0.00). This stems from the differences in eight size-free variables (Tukey Tests, *p* < or = 0.05): PW2, NW, FW1 (male-biased) PTL, MXL, MD2L, FMDB and CQL (female-biased). However, all the differences were very small (Table 4). For each of these variables, at least one of the Cohen’s method assumptions was satisfied and the effect size was rather low (d ≤ 0.31).

**Table 4:** Effect Size based on Cohen’s d coefficient for statistically significant differences (posthoc, MANOVA) for sexually dimorphic skull measurements of *Aiypysurus eydouxii*. The values marked bold indicate for which character the assumption of normality and homogeneity met with < 0.1.

Character	Normality test	Homogenity test	Cohen d coefficient
PW2	*p* = 0.00	***p* = 0.93**	−0.27
NW	*p* = 0.00	***p* = 0.86**	−0.24
FW1	*p* = 0.00	***p* = 0.97**	−0.21
PTL	*p* = 0.00	***p* = 0.4**	0.23
MXL	*p* = 0.03	***p* = 0.58**	0.13
MD2L	***p* = 0.09**	*p* = 0.06	0.26
FMDB	*p* = 0.00	***p* = 0.39**	−0.21
CQL	*p* = 0.01	***p* = 0.18**	0.31

### PCA on Burnaby corrected variables

**Figure 3:**
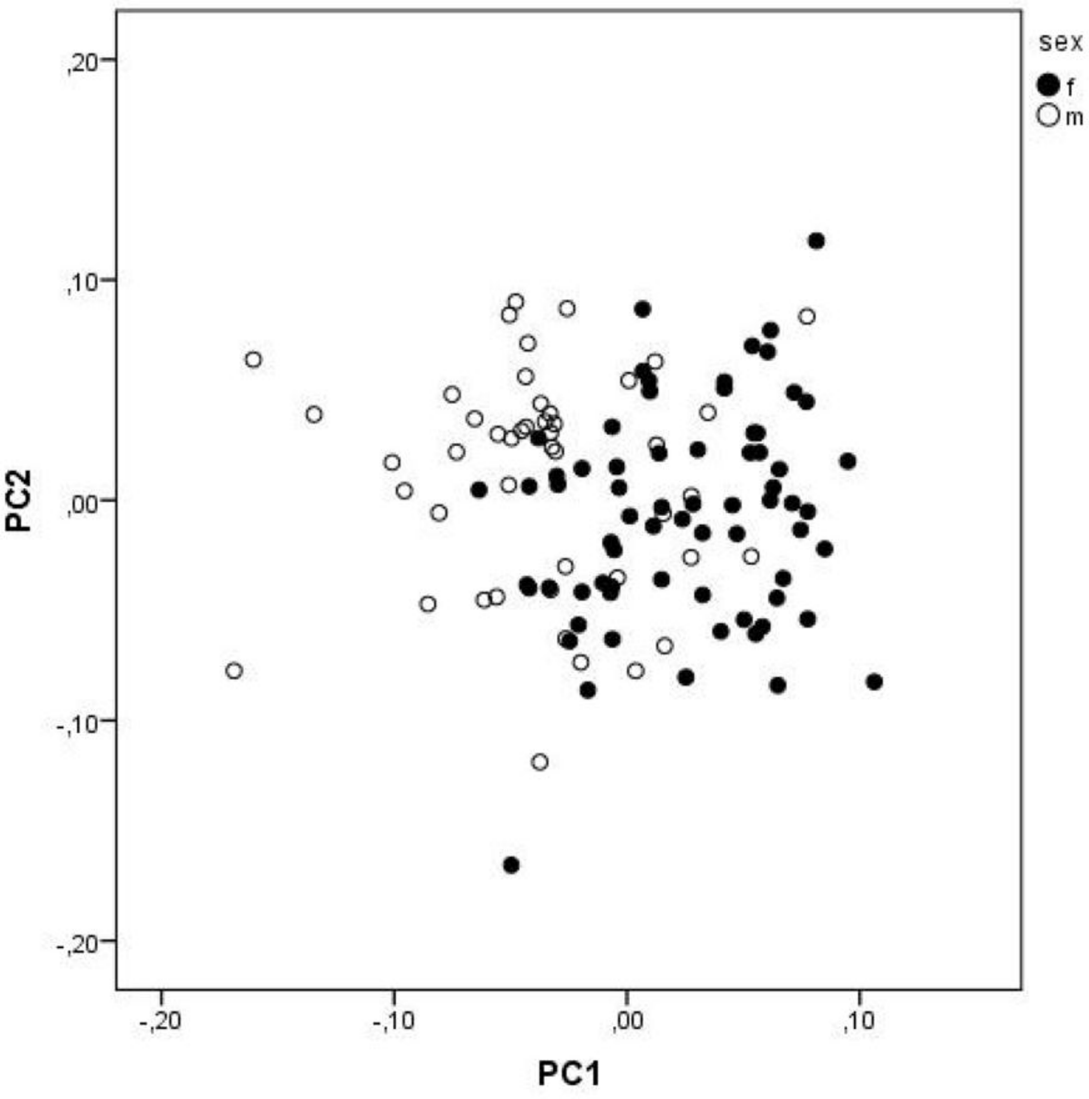
A scatterplot of two first PC from PCA. A scatterplot of two first principal components from the principal component analysis of size-free variables (Burnaby correction) from skull measurements of *Aipysurus eydouxii*.

The first three PCs explain 35.29% of the total variation. The characters that contribute the most (loadings above 0.5) to the PC1 are FW1, FW2, FMDB and CQL, and to PC 2 are FMDB, ECT and PFH (Table 5). However, the separation of sexes is minimal, and both groups largely overlap (Fig. 3).

**Table 5:** Variable loadings for PCA of size-free variables.

Variable	PC 1	PC 2	PC 3
SH	−0.104912	−0.032459	0.218550
SW	−0.069266	0.065903	0.110368
PW1	−0.213465	−0.166428	0.005246
PW2	−0.206810	−0.130372	−0.056481
PL	−0.040639	0.046803	−0.091790
NCL	−0.032124	−0.316628	−0.113869
NL	−0.040150	0.170144	−0.433360
NW	−0.181976	−0.022397	−0.255104
FL	0.005978	−0.255888	0.011502
FW1	**−0.611805**	−0.034259	0.053263
FW2	**−0.664654**	−0.028120	−0.064756
PLL	0.357289	−0.028188	0.348687
PTL	0.495805	−0.072554	−0.324576
PTTL	0.299879	0.136446	−0.785151
PRART	0.047956	0.044871	0.185875
MXL	0.367531	−0.284565	−0.013929
MDL	0.219339	0.073508	0.280331
MD2L	0.295461	−0.056536	0.281903
DENT	0.244811	−0.300780	0.110874
FMDB	**−0.670745**	**0.586936**	−0.162348
ECT	0.245511	**0.656727**	0.396764
QL	0.226299	−0.065300	0.047341
CQL	**0.633311**	−0.279305	0.060596
PFL	−0.470518	**−0.567637**	0.189034
PFH	0.095051	0.463973	0.274697
STP	0.219201	−0.053230	0.130038

**Note:**

Variable loadings for principal components analysis size-free variables (Burnaby correction) from skull measurements of *Aipysurus eydouxii*. Variables that load strongly on PC (|>0.5|) are bolded. For explanation of acronym, see Table 1.

### Allometry

The multivariate allometric coefficients are given in Table 6. The overall pattern of skull growth is similar in both sexes: SW, NL, NW, FW1, PLL, MXL, QL and PFL growth with positive allometry in respect to overall skull size and SH, PW1, PW2, PL, FL, PTL, PRART, MDL, DENT and CQL growth with negative allometry. A few characters show opposite allometric patterns in males and females. These are FW2, PTTL, ECT and STP, which show positive allometry in males and negative in females, and MD2L, FMDB and PFH for which the reverse trend is observed. The differences between male and female multivariate allometric coefficients are of small magnitude (<0.1) for nine characters, and for another nine characters, these differences are between 0.2–0.76. Six characteristics were found to be statistically different between the sexes, which suggests that these differences, although subtle, may result from growth allometry. An another dimorphic distances are PTL that growths isometrically in females (0.99) and with negative allometry in males (0.83), showing moderate difference in allometric coefficients between males and females (0.16), and NW that growths with almost identical allometry in both sexes (differences = 0.03), what suggest, that differences between the sexes are constant during ontogeny.

**Table 6:** Multivariate allometric coefficients for males and females of *Aipysurus eydouxii*.

	Females	Males
SH	0.66	0.71
SW	1.11	1.17
PW1	0.88	0.92
**PW2**	**0.39**	**0.61**
PL	0.64	0.75
NCL	1.24	1.22
NL	1.22	1.28
**NW**	**1.07**	**1.10**
FL	0.95	0.89
**FW1**	**1.21**	**1.45**
FW2	0.79	1.43
PLL	1.20	1.10
**PTL**	**0.99**	**0.83**
PTTL	0.87	1.06
PRART	0.87	0.70
**MXL**	**1.41**	**1.07**
MDL	0.99	0.83
**MD2L**	**1.07**	**0.80**
DENT	0.81	0.89
**FMDB**	**1.15**	**0.39**
ECT	0.97	1.45
QL	1.32	1.29
**CQL**	**0.95**	**0.71**
PFL	1.44	1.34
PFH	1.04	0.86
STP	0.78	1.16

**Note:**

Multivariate allometric coefficients for males and females of *Aipysurus eydouxii*. The characters that were shown to be sexually dimorphic (see the text) are bolded.

The bivariate allometric coefficients are given in Table 7. The distances have low correlation and determination coefficients, which reflect high variability in these traits.

**Table 7:** Linear allometry results for males and females of *A. eydouxii*.

Baseline	Character	Sex	Intercept	Intercept 95% Confidence intervals		Slope	Slope 95% Confidence intervals		R^2^
SVL	BW	F	−5.591	−7.929	−3.254	2.839	2.012	3.667	0.698
		M	−9.912	−13.283	−6.542	4.415	3.189	5.64	0.566
	SL	F	−0.6208	−1.238	−0.004	0.663	0.445	0.881	0.502
		M	−0.9108	−1.332	−0.4891	0.778	0.6244	0.931	0.781
	SH	F	−0.8219	−1.393	−0.2509	0.544	0.3426	0.7463	0.368
		M	−1.186	−1.665	−0.7167	0.686	0.5151	0.8564	0.651
	SW	F	−1.1	−1.898	−0.3023	0.728	0.4456	1.01	0.348
		M	−2.125	−2.822	−1.428	1.11	0.8652	1.363	0.706
SL	SH	F	−0.397	−0.5958	−0.1984	0.886	0.7306	1.05	0.462
		M	−0.3707	−0.5606	−0.1807	0.871	0.7165	1.025	0.665
	SW	F	−0.6635	−0.884	−0.443	1.295	1.118	1.472	0.692
		M	−0.7186	−1.02	−0.4176	1.339	1.094	1.583	0.657
	PW1	F	−0.5037	−0.7342	−0.2732	1.116	0.9307	1.301	0.54
		M	−0.5264	−0.8171	−0.2357	1.14	0.9036	1.376	0.537
	PW2	F	−0.3941	−0.6718	−0.1164	0.968	0.745	1.191	0.112
		M	−0.1266	−0.3255	0.0723	0.722	0.6006	0.9238	0.516
	PAR	F	−0.4271	−0.6703	−0.1838	1.031	0.8358	1.226	0.399
		M	−0.3511	−0.5925	−0.1096	0.974	0.7778	1.17	0.563
	NCL	F	−0.9758	−1.257	−0.6951	1.405	1.179	1.63	0.569
		M	−1.089	−1.436	−0.4725	1.496	1.214	1.777	0.618
	NL	F	−2.204	−2.864	−1.544	2.339	1.809	2.868	0.14
		M	−1.299	−1.657	−0.9412	1.615	1.324	1.905	0.656
	NW	F	−1.418	−1.846	−0.9907	1.62	1.276	1.963	0.248
		M	−1.136	−1.544	−0.7276	1.415	1.083	1.746	0.408
	FL	F	−1.185	−1.521	−0.8486	1.428	1.159	1.698	0.403
		M	−0.8507	−1.152	−0.5495	1.157	0.912	1.401	0.518
	FW1	F	−1.517	−1.951	−1.084	1.761	1.412	2.109	0.345
		M	−1.569	−2.078	−1.061	1.821	1.408	2.235	0.446
	FW2	F	−0.9189	−1.24	−0.598	1.289	1.031	1.547	0.331
		M	−1.551	−2.069	−1.033	1.806	1.385	2.227	0.415
	PLL	F	−1.424	−1.824	−1.024	1.722	1.401	2.043	0.452
		M	−1.096	−1.419	−0.7726	1.453	1.19	1.716	0.672
	PTL	F	−0.6693	−1.003	−0.3357	1.358	1.09	1.626	0.359
		M	−0.3804	−0.6573	−0.1034	1.115	0.8899	1.34	0.576
	PTTL	F	−1.434	−1.992	−0.8753	1.905	1.456	2.353	0.086
		M	−1.299	−1.918	−0.6804	1.793	1.291	2.296	0.154
	PRETR	F	−1.745	−2.228	−1.262	1.687	1.299	2.075	0.141
		M	−1.035	−1.387	−0.6827	1.116	0.8305	1.403	0.293
	MXL	F	−1.492	−1.897	−1.087	1.867	1.542	2.192	0.507
		M	−1.132	−1.596	−0.6672	1.568	1.191	1.946	0.39
	MDL	F	−0.3384	−0.6314	−0.04529	1.282	1.047	1.517	0.453
		M	0.05617	−0.114	0.2263	0.964	0.8254	1.102	0.778
	MD2L	F	−0.4453	−0.7266	−0.164	1.304	1.079	1.53	0.514
		M	−0.0152	−0.176	0.1455	0.953	0.8227	1.084	0.798
	DENT	F	−0.7788	−1.156	−0.4017	1.36	1.058	1.663	0.184
		M	−0.5684	−0.8579	−0.2789	1.187	−0.9517	1.422	0.577
	FMDB	F	−2.091	−2.692	−1.491	2.124	1.642	2.606	0.165
		M	−1.53	−2.156	−0.9031	1.716	1.207	2.225	0.052
	ECT	F	−1.939	−2.577	−1.302	2.188	1.676	2.701	0.151
		M	−1.788	−2.37	−1.205	2.063	1.59	2.537	0.434
	QL	F	−1.494	−1.882	−1.107	1.698	1.387	2.009	0.438
		M	−1.429	−1.754	−1.105	1.644	1.38	1.908	0.742
SL	CQL	F	−1.2	−1.546	−0.8533	1.395	1.117	1.674	0.335
		M	−0.9784	−1.348	−0.609	1.194	0.8941	1.494	0.369
	PFH	F	−1.793	−2.261	−1.325	1.867	1.491	2.243	0.331
		M	−1.586	−2.155	−1.016	1.709	1.246	2.172	0.209
	PFL	F	−2.518	−3.102	−1.934	2.263	1.795	2.732	0.293
		M	−2.207	−2.778	−1.635	2.025	1.56	2.489	0.433
	STP	F	−0.887	−1.199	−0.5766	1.247	0.9972	1.497	0.328
		M	−1.355	−1.8	−0.9104	1.617	1.255	1.978	0.461

**Note:**

Intercepts, slopes and their 95% confdence intervals of RMA-regression of log-transformed skull measurements on SVL and SL of male and female *Aipysurus eydouxii*.

In general, both sexes follow similar trajectories; however, there are some differences too. Although SL and SH scale with negative allometry in respect to SVL in both sexes, the skull width SW scales with positive allometry in males (when scaled against the SL, these differences disappear). Other distances that strongly differ in the growth pattern when scaled against SL are nasal length (NL), retroarticular process, mandible length (MD2L), and mandibular fossa length (FMDB) that are female-biased and frontal width (FW2), which is male-biased. Because the MD2L to FMDB ratio reflected the in-lever to out-lever when the jaw adducted and these distances scale differentially in both sexes, which may lead to dimorphism in the mechanical advantage (Vincent et al., 2007). However, when ANCOVA was performed with FMDB as the dependent variable, sex as a categorical factor, and MD2L as a continuous variable, there was no sex-based differences in mechanical advantage (F = 2.416, *p* = 0.119).

## Discussion

### Sexual size dimorphism

In many snake species, female snakes have longer bodies compared to conspecific males (Shine, 1993) and there is a positive correlation between female body size and the number and size of their offspring or clutches (Madsen & Shine, 1994; Rivas & Burghardt, 2001). Moreover, the offspring of larger females have a higher rate of survival (Ford & Seigel, 1989; Rivas & Burghardt, 2001), which further favours an increased female body size. In our study, female *A. eydouxii* were larger than males (however, we are cautious it may be partially biased by sample size). Surprisingly, statistical analysis showed that females were not heavier than males, which means that despite being longer, they remain slender-bodied like males, an unusual pattern among snakes (Shine, 1993) including close relatives of *A. eydouxii*, such as *A. laevis* (Burns & Heatwole, 2000). However, *A. eydouxii* is a fish-egg feeder, and such prey often hides in small crevices, under reef stones, and other hard to access places. Thus, a slender body shape may be an advantage in browsing for food and help to compensate for higher female energy demands (see below).

### Skull size dimorphism

In many species, an increase in body size is accompanied by a relative enlargement of the head, which helps the animal to explore a broader prey spectrum. It is thought to be one means of compensating for greater energy expenditures (maintaining larger bodies and, in the case of females, providing nutritional components for developing embryos) (e.g. Elgee & Blouin-Demers, 2011; Borczyk, 2015). Females grow larger in approximately two thirds of snake species studied so far and usually females possesses larger heads (Shine, 1993). These differences can be a result of a disproportional (allometric) head growth pattern, as exemplified by *Laticauda colubrina* (Shetty & Shine, 2002), or are constant during ontogeny, like in *Natrix natrix* (Borczyk, 2015). However, this is not the case in *A. eydouxi*. In this species, the smaller sex (males) has relatively longer, higher, and wider heads, which stands contrary to general predictions.

Differences in head size and shape often result from selective pressure on dietary/feeding niche separation, as they are usually accompanied by differences in average prey size, type, or both (e.g. Shine, 1986, 1991, 1993; Houston & Shine, 1993; Forsman & Shine, 1997; Keogh, Branch & Shine, 2000; Shine et al., 2002; Gregory & Isaac, 2004; Vincent, Herrel & Irschick, 2004; Shine et al., 2012; Borczyk, 2015). The species in this study is one of the very few snake species that feed on fish eggs. The egg diameter of reef fishes eaten by this species is below the potential prey size any macrostomatan snake can swallow: Shine et al. (2004) found that *Emydocephalus annulatus*, a closely related species and obligatory fish-egg feeder, eats fish eggs of diameter less than 1 mm, and this prey size may be safely extrapolated to *A. eydouxii*. Thus, such dietary habits do not leave space for food niche divergence in terms of prey size, as in *A. eydouxii*, prey size does not predict gape size (head size). The head size decreases proportionally in the larger sex, suggesting that females invest more energy in body cavity growth at the cost of head size. Simultaneously, a small head seems to be an advantage for food seeking in difficult-to-access places, similarly as the slender body. A similar pattern is observed among other hydrophiinae species that prey on burrowing eels and a microcephalic forebody-slender body form in snakes evolved at least nine times independently within hydrophiine radiation (Sherratt, Rasmussen & Sanders, 2018; Sherratt et al., 2019a; Sherratt et al., 2019b; Hampton, 2019). Since both sexes exploit the same resources, the smaller head size of females may be associated with a decrease in intra-specific competition for food because females may be able to penetrate crevices of diameter too small for males’ heads. On the other hand, if competition is not an issue, females may be able to compensate for higher energy demands by collecting food from a larger number of sources, including those inaccessible to males.

One could speculate that such a dimorphism would give larger-headed males an advantage over smaller-headed ones when mating (or via male-male interaction or female preferences), as in many lizard species (e.g. Borczyk et al., 2014; Gvozdik & Van Damme, 2003; Van Damme et al., 2008). However, head size in snakes does not appear to be important for reproductive behaviour, at least in the male-male competition context (Shine, 1991). Instead, the head also houses the sensory organs, namely the eyes, and recent observations on sea-snake mating behaviour shows that males use visual cues to assess potential sexual partners (Shine, 2005). It has been also shown, that in some snakes species, there is sexual dimorphism in the eye size (Faiman et al., 2018). However, there is no clear pattern, and in some species, such differences are female-biased and in others, male-biased, with different degrees of magnitude (Faiman et al., 2018). This phenomenon remains largely unstudied in snakes, but it cannot be excluded, because there would be a need for more space for the eyeball in one sex (Camilleri & Shine, 1990). In our study, we have found some differences in skull bone proportions in the orbital region (FW2: frontal width at the fronto-parietal suture); however, these differences were quite small.

### Skull shape dimorphism

There are very few reports on sexual dimorphism in skull shape among snakes (e.g. Camilleri & Shine, 1990; Andjelković, Tomović & Ivanović, 2016; Murta-Fonseca et al., 2019; Sherratt et al., 2019b). However, head shape dimorphism is relatively well studied (e.g. Vincent, Herrel & Irschick, 2004; López, Manzano & Prieto, 2013; Borczyk, 2015; Jestrzemski & Kuzyakova, 2018 and others). In the present study, we show that male and female skulls are mostly uniform. The only characteristic that differ between the sexes are nasal width (NW), frontal width (at the fronto-parietal suture, FW1), parietal width (PW2), and mandibular fossa length (FMDB), which are male-biased, and quadrate crest length (CQL), pterygoid length (PTL), mandible length (from the tip to the quadrate articulation: MD2L), and maxilla length (MXL) which are female-biased. Other authors also noted sex differences in the braincase, maxillary, pterygoid, nasals, frontals, supratemporals, and mandible bones in *Acrochordus arafurae*, *Pseudechis porphyriacus*, *Xenodon neuwiedii*, *Natrix natrix* and *N. tessellata* (Camilleri & Shine, 1990; Andjelković, Tomović & Ivanović, 2016; Murta-Fonseca et al., 2019). The dimorphic bones and the direction of dimorphism often differ between species. For example, there is shape dimorphism in pterygoid in *N. tessellata* but not in the closely related *N. natrix* and the reverse is true for the nasal bone (Andjelković, Tomović & Ivanović, 2016). The maxillary and pterygoid bones are larger in females in *X. neuwiedii* (Murta-Fonseca et al., 2019), whereas the reverse is true for *A. eydouxii* (but note some methodological differences–geometric morphometry vs linear morphometry).

Mandible and quadrate bones contribute to gape size (Hampton & Moon, 2013); however, as discussed before, the selective pressure to increase gape size is unlikely in the species studied here. Similarly, pterygoid and maxillary bones are important in prey transport (see Jackson, Klay & Brainerd, 2004), but the fish egg should not pose any difficulties for a macrostomatan snake, and *A. eydouxii* is supposed to be a suction-feeder (McCarthy, 1987). The throat movements during feeding suggesting suction has been observed in *Emydocephalus annulatus*, a closely related fish egg-eater (Goiran, Dubey & Shine, 2013), and taking the derived throat musculature (McCarthy, 1987) and similar prey spectrum may be safely extrapolated to *A. eydouxii*. Instead, differences in skull shape may echo the phylogenetic history of the species and be ancestral characteristics for sea snake radiation since the closest relatives to *A. eydouxii* are macrophagous and feed primarily on fishes (e.g. Voris & Voris, 1983, Sherratt, Rasmussen & Sanders, 2018; Sherratt et al., 2019a). In those species, the females usually eat larger prey items, and thus, the relative size of the structures responsible for prey transport and increased gape size may be ancestrally female-biased. On the one hand, our results may be interpreted as indirect support for prey-size divergence being a factor driving skull dimorphism (Camilleri & Shine, 1990), as species in which the sexes do not differ in prey size also show only subtle or no differences in skull morphology. However, more studies on the relationship between intrasexual diet divergence and skull dimorphism covering a wide range of taxa are needed for testing this hypothesis.

### Allometry

The pattern of skull growth allometry is complex. In general, *A. eydouxii* males showed higher allometric coefficients for distances related to skull width, whereas females showed slightly higher coefficients for distances parallel to the body axis. However, for most cases, this is not reflected in the statistically significant differences in skull shape. Comparing to published data on skull growth in different snake species, it seems that the overall pattern of skull growth is consistent with those described for other species (Rossman, 1980; Hampton, 2014; Hampton & Kalmus, 2014). However, *A. eydouxii* is a member of a highly specialised snake group, marine radiation within Hydrophiinae (Sanders et al., 2013), and its skull form differs from its terrestrial relatives (Young, 1987). Unfortunately, there is no data on it close terrestrial relatives to infer how allometric trajectories in skull growth has changed during terrestrial to marine radiation.

There were low r^2^ values in RMA regression analysis for *A. eydouxii*. In other snake species (Borczyk, 2019 and Borczyk in preparation), the r^2^ coefficients were much higher (i.e., r^2^ between 0.6 and 0.99 for some traits; usually >0.75). It is possible that low correlation and determination coefficients reflect a relaxed selection on the efficiency of large food item manipulation. Small food particles (fish eggs) do not pose the same kind of problems as larger prey, such as swallowing whole fish, whose diameter may be equal to or bigger than the head diameter. The intraoral prey transport in *A. eydouxii* seems to rely more on suction (McCarthy, 1987; Goiran, Dubey & Shine, 2013) than on so-called pterygoid walk (Boltt & Ewer, 1964; Jackson, Klay & Brainerd, 2004). Thus some traits may exhibit a much greater level of interspecific variation without decreasing the feeding efficiency of an individual–a reverse trend of truly macrophagous species, where high integration of the skull is key for effective intraoral prey transport.

## Conclusions

Our results show that females attain longer bodies than males, which stands in agreement with the general pattern observed among different snake species. However other features analysed in the present research did not follow the most predictable pattern. Females, being the sex with possibly higher energy demands, are expected to exhibit higher food intake, which, in many species, is associated with a thicker body and larger head, allowing ingestion of larger prey. However, the high feeding specialization of *A. eydouxii* does not leave space to increase body thickness and head size above a certain threshold. Thus it seems that high feeding specialization may serve as a strong selective force that equalizes or extends the slope of the variation in opposite way than most predictable. The present study work may be a valuable contribution to future research based on rather atypical life strategies to investigate how certain trade-offs may shape sexual dimorphism.

## Supplemental Information

10.7717/peerj.11311/supp-1 Supplemental Information 1 List of skull measurements of males and females of *Aipysurus eydouxii*.

10.7717/peerj.11311/supp-2 Supplemental Information 2 List of studied specimens of *Aipysurus eydouxii*.

## Additional Information and Declarations

### Data Availability

The following information was supplied regarding data availability: Data are available in the Supplemental Files.
