# Supplementary material for: Sexual dimorphism and skull size and shape in the highly specialized snake species, Aipysurus eydouxii (Elapidae: Hydrophiinae)
Source: PeerJ. 2021 Apr 20;9:e11311. doi: 10.7717/peerj.11311 (PMC8063874; doi:10.7717/peerj.11311)
Supplement: Supplemental Information 2 [file peerj-09-11311-s002.docx]

Appendix 1. List of specimens used in the study. All skulls are from the collection of Field Museum of Natural History (FMNH). An Asterix (*) denotes specimens with no data on their SVL or body weight.

FMNH 231613, 231614, 231015, 231616, 231617, 231618, 231619, 231620, 231621, 231622, 231623, 231624, 231625, 231626, 231627,231628, 231630, 231631, 231632, 321633, 231634, 231635, 231636, 231637, 231709, 231711, 232775, 245579*, 245580*, 245581*, 245583*, 245584*, 245585*, 245586*, 245587*, 245590*, 245591*, 245593*, 245596*, 245597*, 245598*, 245600*, 245601*, 245603*, 245605*, 245606*, 245607*, 245608*, 245609*, 245612*, 245614*, 245615*, 245616*, 245617*, 245631*, 245618*, 245619*, 245620*, 245621*, 245622*, 245623*, 245624*, 245625*, 245626*, 245627*, 245628*, 245629*, 245630*, 245632*, 245633*, 251467*, 251469*, 251471*, 251473*, 251474*, 251475*, 251476*, 251477*, 251478*, 251479*, 251480*, 251482*, 251484*, 245634*, 245635*, 245637*, 245638*, 251450*, 251453*, 251456*, 251459*, 251460*, 251464*, 251466*, 232776, 233179, 233179, 233180, 233182, 233184, 233187, 233188, 233189, 233190, 233191, 233192, 233193, 233194, 233196, 245569, 245572, 245575, 245576, 245577, 245578
